# Research on Vehicle Lane Change Warning Method Based on Deep Learning Image Processing

**DOI:** 10.3390/s22093326

**Published:** 2022-04-26

**Authors:** Qiang Zhang, Ziming Sun, Hong Shu

**Affiliations:** 1 Department of Automotive Engineering, College of Mechanical and Vehicle Engineering, Chongqing University, Chongqing 400044, China; zhangqiang2@caeri.com.cn (Q.Z.); circle2022@outlook.com (Z.S.); 2 State Key Laboratory of Vehicle NVH and Safety Technology, China Automotive Engineering Research Institute Co., Ltd., Chongqing 401122, China

**Keywords:** vehicles, vehicle detection, deep learning, lane-changing detection, Mask R-CNN

## Abstract

In order to improve vehicle driving safety in a low-cost manner, we used a monocular camera to study a lane-changing warning algorithm for highway vehicles based on deep learning image processing technology. We improved the mask region-based convolutional neural network for vehicle target detection. Suitable anchor frame ratios were obtained by means of K-means++ method clustering for 66,389 vehicle targets with the width/height ratio, which is one more set of anchor frames than the original setting, so as to ensure that the generation accuracy of candidate frames can be improved without sacrificing more network performance. Using the vehicle target annotation set, we trained the vehicle targets. Through the analysis of indicators for mean average precision, a new set of anchor frames was added to improve the accuracy of vehicle target detection. Based on the improved vehicle detection network and an end-to-end lane detection network in series, we proposed an algorithm for the detection of highway vehicle lane-changing behavior with the first-person perspective by summing the inter-frame change rates in the vehicle lane-changing data pool. After the identification and verification of the marked lane-changing picture sequences, a lane-changing detection accuracy rate of 94.5% was achieved.

## 1. Introduction

In recent years, the technology of advanced driver assistance systems has been rapidly developed and applied. However, the current functions of advanced driver assistance systems configured by vehicles on the road are uneven, and some lack basic driving assistance functions, such as collision avoidance warning and lane-changing warning functions. In addition, 18% of road traffic accidents and 10% of delays are caused by unsafe lane-changing behavior in China [1]. A large proportion of straight driving sections under highway conditions and long-term driving under good road conditions will make the driver fatigued and more likely to cause an accident. Therefore, it is beneficial to improve vehicle safety by the early warning of lane-changing behavior of the preceding vehicles. How to improve the driving safety of these vehicles in a low-cost manner and realize the development of the corresponding functions of the driving warning system have important research value. Due to the low-cost and easy deployment of the monocular camera, it can complete the detection and positioning tasks of all targets in the detection module of the warning system. Therefore, based on a monocular camera, we used deep learning image processing technology to research and develop the vehicle lane-changing warning system. The warning system realized perception of the surrounding environment by a low-cost monocular camera. The basic environmental information of the road should include vehicle information and lane information. Through the deep learning pre-processing module, the information of preceding vehicles and the lane information of the ego-vehicle were obtained and combined with the analysis of a lane-changing warning post-processing module to obtain warning information of dangerous road conditions, which improve the driving safety of the ego-vehicle.

Due to the parallel computing wave triggered by deep learning in recent years, a large number of algorithms based on deep learning methods have emerged in the field of target detection. Region-based Convolutional Neural Networks (R-CNNs) were proposed by Girshick et al., in 2014, which combines the region proposal with the image feature convolutional neural network to improve the detection accuracy [2]. The proposed region size was re-adjusted and used as the input of the standard CNN architecture [3], such as Alexnet [4], Visual Geometry Group Network (VGG), Inception, and Deep Residual Network (ResNet). Among them, the last layer of the CNN network architecture was trained using the Support Vector Machine (SVM) algorithm to detect whether there is a target of one of the target classes in the region. According to a linear regression model, the bounding box obtained by the region proposal could further converge to the real bounding box. However, the R-CNN method did not achieve the end-to-end learning.

In 2015, Girshick et al. proposed an improved algorithm for R-CNN—Fast R-CNN [5], which only performs CNN inference once, greatly reducing the amount of calculation. A new pooling technology, Region of Interest (RoI) pooling, was introduced. According to the regional suggestions, CNN features were pooled together. In this way, after the reasoning of the CNN network, the features obtained in the pooling step would form a set. The advantage of this approach was that the end-to-end learning can be achieved, thus avoiding the use of multiple classifiers. The previous SVM classifier was replaced by a Softmax layer, but still required a lot of computing power because it still proposes a selective search area.

In 2016, Ren et al. proposed Faster R-CNN [6], which replaces selective search with a CNN framework such as VGG and Inception. A Region Proposal Network (RPN) was proposed. RPN is a fully convolutional network that can achieve the end-to-end learning. The most important breakthrough was to make the RPN network and the object detection network share the convolutional features, which realizes the area proposal with almost no computational cost and reduces the computational time.

Mask R-CNN was proposed by He et al., in 2017 [7], and made two improvements on the basis of Faster R-CNN. The RoI pooling layer was replaced with an RoI align layer, and a mask branch was added to predict the segmentation mask of each region of interest. Because the RoI align layer used bilinear interpolation, the output of RoI was aligned with the source image, resulting in more accurate instance segmentation. Because the mask branch was operated in parallel with the original classification branch and the border regression branch, time loss was reduced and the segmentation accuracy was improved.

In terms of research on algorithms for detecting lane-changing behavior, there is a method based on a third-person fixed perspective. By detecting the angle between the trajectory of the target vehicle and the fixed lane line, when the angle is greater than a certain threshold, it is determined that the target vehicle has changed lane. Shi et al. [8] calculated the variance of the lateral distance between the target vehicle’s trajectory line and the corresponding points of the ego-vehicle’s lane line based on the third-person fixed perspective, and determined the lane-changing behavior based on this. Although it is possible to send lane-changing warning information to the driver of the ego-vehicle through third-party observation and through the internet of vehicles, it requires a large number of cameras beside the road, and the threshold for drivers to use is relatively high. Hu [9] proposed an algorithm for extracting the edge feature of the lane line with a direction-tunable filter, using a Kalman filter to track and correct the parameters of the lane line, and proposed a vehicle lane-changing detection method based on the lane. Wei et al. [10] proposed a lane-changing detection method for vehicle violation based on the actual behavior of the vehicle crossing the solid line. Zhao et al. [11] proposed a vehicle merging warning system with a preset safety zone. When the midpoint of the vehicles on both sides of the ego-vehicle lane crossed the trapezoidal safety zone of the ego-vehicle, the warning message was prompted. The above methods all determined the result of the lane-changing, failed to meet the requirement of real-time warning to a large extent, and could not feedback in a timely manner the lane-changing information of the preceding vehicles.

A good prediction for the lane-changing actions of surrounding vehicles will significantly improve the safety and passenger comfort of driver-assisted and automated vehicles [12]. Woo et al. [13] installed a position sensor and six lidars on the ego-vehicle to obtain the positions and speeds of four adjacent target vehicles, used SVM to estimate the driver’s intention, and predicted the target vehicle’s trajectory to detect lane-changing behavior. Zhang et al. [14] used the relative speeds and distance data of the target vehicle and four adjacent vehicles to simulate the lane-changing behavior of the target vehicle with a continuous hidden Markov model, calculated the lane-changing probability, and predicted the lane-changing behavior of the target vehicle, which reached 85% of the true positive rate. Zhang et al. [15] used a passive-aggressive algorithm to design a lane-changing source classifier with a large number of lane-changing operations as training samples according to the motion state of the target vehicle before the lane change and the parameters of the relative motion with the surrounding vehicles, and proposed an online transfer learning strategy to predict the lane-changing intention of the target vehicle. This method requires an ego-vehicle equipped with a GPS and millimeter-wave radar. Based on the data provided by radars and cameras, Lee et al. [16] input a simplified bird’s-eye view into a convolutional neural network to predict the lane-changing intention of the target vehicle. Wei et al. [17] proposed a deep residual learning neural network to identify the lane-changing behavior of the vehicle on a highway based on images captured by a forward-facing vehicle camera and measured ego-vehicle braking and accelerator pedal forces, speed, steering angle, and longitudinal and lateral acceleration information, achieving an accuracy of 87%.

In summary, the research on the vehicle lane-changing warning algorithm is partly based on the determination of the lane-changing result, which leads to the inability to feedback in a timely manner the lane-changing information of the preceding vehicles, or is based on the lidar or the radar and camera to obtain the relative motion parameters between the ego-vehicle and the target vehicles, which leads to high hardware cost, and the recognition accuracy needs to be further improved. In order to realize the lane-changing warning of the ego-vehicle in a low-cost way to improve the safety of the driver assistance system, we studied the lane-changing warning algorithm based on a monocular camera. The current mainstream target detection frameworks such as Mask R-CNN are multi-target detection networks, which are not optimized for single target detection tasks such as vehicle detection. We improved the generation process of the candidate frame of Mask R-CNN by counting the relevant size parameters of the vehicles, so as to obtain a better candidate frame and improve the detection accuracy of the target vehicles. We proposed to calculate the sum of the inter-frame change rate when the target vehicle changes lane based on the first-person perspective. We could remind the driver in a timely manner to pay attention to dangerous lane-changing behavior by determining the target vehicle’s lane-changing behaviors instead of the lane-changing results, and we achieved a high lane-changing detection accuracy.

## 2. Materials and Methods

### 2.1. Improved Network Framework of Vehicle Target Detection

In the field of deep learning, there are many ways to obtain the bounding box of the vehicle target. One method is to directly predict the bounding box value of the vehicle. Szegedy et al. [18] proved that neural networks can be directly used for coordinate regression purposes. Yolo V1 [19] directly predicted the four values of the bounding box, but the corresponding loss function did not truly reflect the accuracy of the predicted box. The disadvantage of this method is that it is more inclined to use a large-size bounding box. Although the approximate position of the vehicle can be identified, it has a large defect in the warning system that requires accurate vehicle positioning. In addition, the training process of this method is unstable, because the predicted frame value may change significantly as the posture of the vehicle changes or the distance relationship changes. Other methods, such as Mask R-CNN, Faster R-CNN, and Single Shot Detector (SSD) [20], use anchors. Based on the preset anchors, after obtaining the proposed frame, going back, and fine-tuning the frame size and position, the problems of former method can be solved.

**Figure 1:**
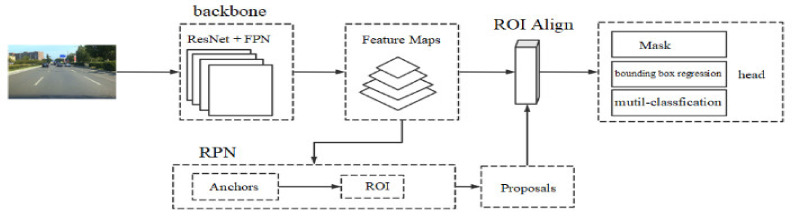
Mask R-CNN network framework.

Mask R-CNN is a multi-task deep learning model. This network can realize the classification and positioning tasks of the instances in the picture by the end-to-end learning, and can realize the pixel-level mask to complete the instance segmentation. Mask R-CNN includes three main sub-networks, namely the backbone network, which is a combination of ResNet and Feature Pyramid Network (FPN) [21], RPN, and the head network, as shown in Figure 1.

The head network has three branches. In the classification and regression branches, the input of the head network first passes through a two-layer convolutional network, enters two fully connected layers, and then enters a Softmax classification layer and the linear regression layer. The same activation function as in the RPN network is used. The Mask branch goes through the steps of multi-layer convolution, Batch Normalization (BatchNorm), Rectified Linear Unit (ReLU) layer, etc., and the 28 × 28 Mask feature map of each class is obtained. The activation function Sigmoid is used to distinguish the value of each pixel as 0 or 1. A binary cross-entropy loss function is used for training.

**Figure 2:**
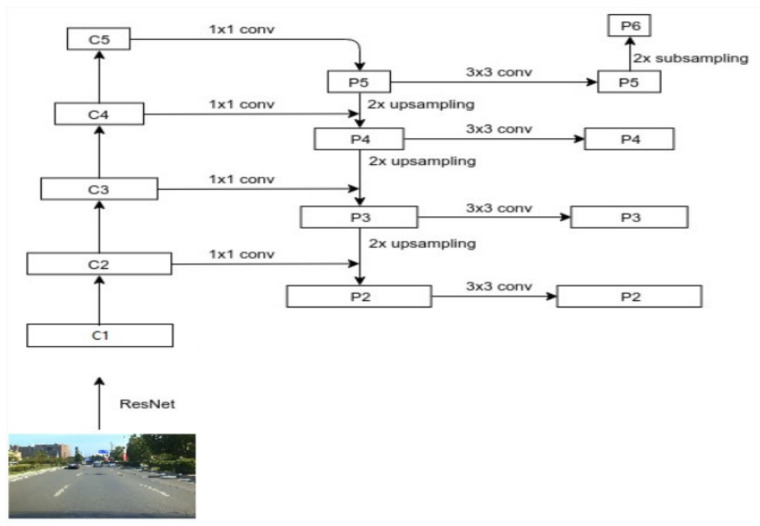
Relationship between Resnet and FPN.

**Figure 3:**
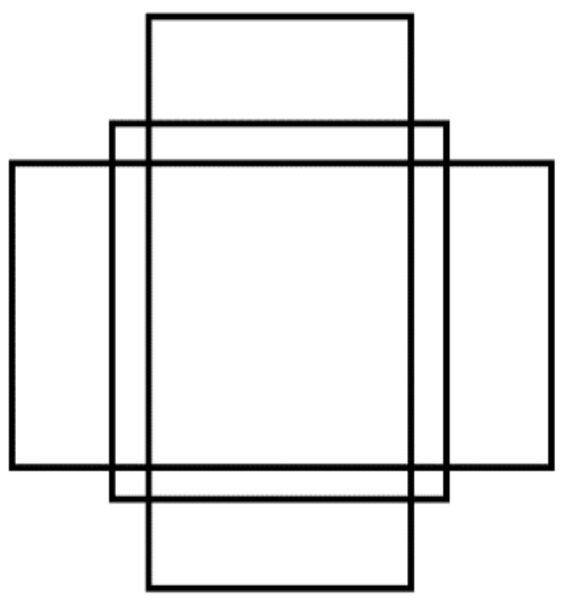
Three ratios of same size anchor.

Anchor boxes are a series of candidate boxes obtained by combining different aspect ratios and different aspect proportions. According to (P2, P3, P4, P5, P6), five pyramid feature layers, three anchor frames, and 15 anchor frame sizes are set in Mask R-CNN to detect the preset of the frames (see Figure 2). There are 5 scales of anchor frames (16, 32, 64, 128, 256). The ratios of anchor frames have three kinds of aspect ratios (0.5, 1.0, 2.0). Three anchor boxes with different aspect ratios, as shown in Figure 3, are generated on each feature map as the pre-selection boxes.

The proportions of the anchor frames need to be modified appropriately to adapt to different target detection tasks, speed up the convergence of the model, and improve the positioning accuracy of the targets. In order to obtain the appropriate anchor frame ratios setting for the vehicle targets, the K-means++ clustering algorithm [22] was performed for the vehicle bounding box label of the dataset to obtain the appropriate width/height ratios of anchor frames.

The K-means algorithm is a distance-based clustering algorithm that uses the distances between points as an indicator of similarity. If the distance between two pixels is closer, the similarity is higher. This algorithm gathers points that are close to each other into a cluster, and finally obtains compact, bounded, and independent cluster objects. The inherent defect of the K-means algorithm is that it needs to manually specify the number of cluster points. The K-means algorithm is more sensitive to the location of the starting point. The K-means++ algorithm compensates for these defects to a certain extent. It can speed up the convergence of the model and improve the positioning accuracy of the target by using K-means++ clustering to obtain more suitable anchor frame ratios of vehicle targets. A higher positioning accuracy is used to meet the need of accurate vehicle lane-changing detection.

We used the road target dataset labeled by CrowdAI Company in the United States to perform the clustering task. The data contained traffic information in Mountain View, California, USA, and nearby cities, including 9423 frames of pictures (sampled at 2 Hz) and a road traffic information dataset for a total of 72,064 targets. The resolution was 1920 × 1200.

We cleaned the dataset before clustering. The dataset contained three different targets: cars, trucks, and pedestrians. The lane-changing warning system we designed was to conduct research on vehicles. Pedestrians were not the subject of research, so the pedestrian tag data were eliminated, leaving 66,389 vehicle targets.

If the anchor was appropriately increased in an appropriate proportion, more appropriate anchor frames could be generated, which could theoretically increase the value of the average Intersection over Union (IoU). This was because choosing more priori anchor frames would cause the anchor frames to have greater overlap with the real frames. However, as the number of anchor frames increases, the corresponding convolution filter would also increase, increasing the size of the network and leading to a longer training time. Therefore, we set the ratios of anchor frames to 4, which is one more set of anchor frames than the original setting to ensure that the generation accuracy of candidate boxes can be improved without sacrificing more network performance.

**Figure 4:**
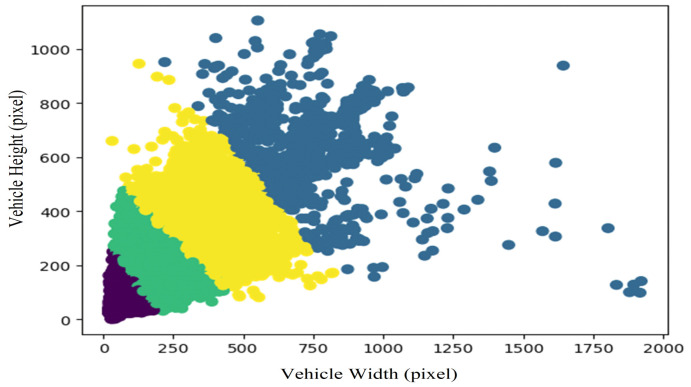
The clusters of four cluster points.

**Figure 5:**
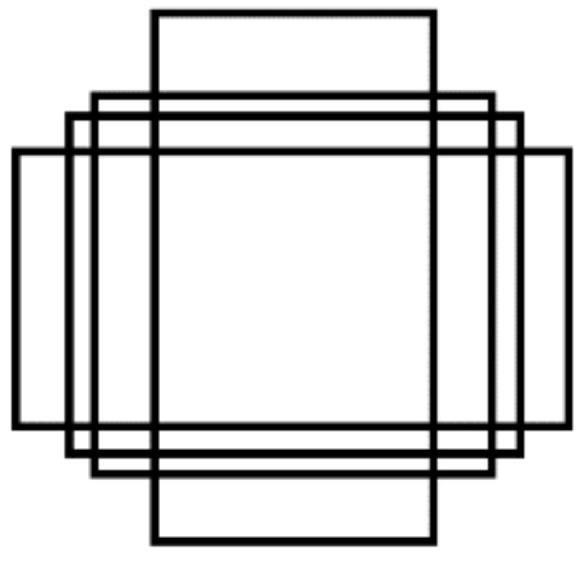
The new anchor ratios.

The results of clustering four cluster points according to the vehicle’s aspect ratio are shown in Figure 4, and the colors distinguish the clusters of the four cluster points. According to the coordinates of the center of the cluster points, the corresponding four anchor frames were set with a ratio of (1.26249845, 1.20118061, 1.40220561, 1.15757855). As the vehicle proportions were relatively consistent, taking the average of the above four types of anchor frames could obtain the new anchor frame ratio of 1.256. Adding a new ratio to the original anchor frame ratios could obtain four anchor frame width/height ratios (0.5, 1.0, 1.256, 2.0). The anchor frames of the feature map would generate four proportions of frames on the original field of view, as shown in Figure 5.

### 2.2. Dataset Preparation for Vehicle Detection Network

The datasets in the field of target detection usually include the Pascal Visual Object Classification (VOC) dataset, MS Common Objects in COntext dataset, and ImageNet dataset. We annotated the original data according to the standard format of the Pascal VOC dataset, and created the vehicle detection dataset of CQ_Vehicle_Dataset for the training of the improved Mask R-CNN network.

**Figure 6:**
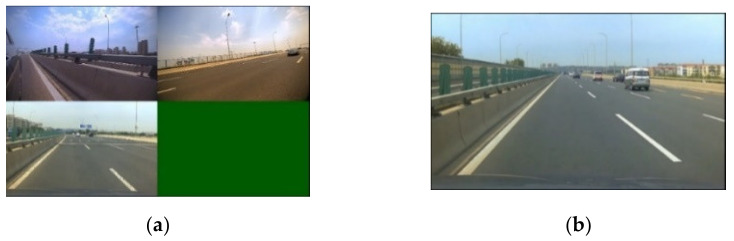
(**a**) Original video screenshot. The upper left indicates the left rear view of the ego-vehicle, the upper right indicates the right rear view, and the lower left indicates the front view; the rear view is not displayed. (**b**) Cropped screenshot of forward video.

The original data were 4-channel videos collected by the staff of the China Automotive Engineering Research Institute. The video contained a total of 4 perspectives, namely the left, the right, the front, and the back perspective. The road information in front of the vehicle and vehicle dynamic information were analyzed and processed, so the data source needed to be tailored. An example of the original video screenshot was shown in Figure 6a. The video screenshot was obtained after cropping by FFmpeg software, as shown in Figure 6b.

The size of the video was 1280 × 720. The 64 videos obtained were subjected to frame extraction processing to obtain a sufficient amount of data. A batch conversion file was written based on Python. The 64 videos in the folder were batch-converted and sampled every 50 frames to prepare pictures as a prerequisite for annotation. After data cleaning, 2537 images were retained, which covered the urban, suburban, and highway driving conditions, and the weather covered sunny and rainy. The size of the image was 1280 × 720 pixels. On this basis, a total of 317 images had been marked with mask information for a total of 1364 vehicle targets.

**Figure 7:**
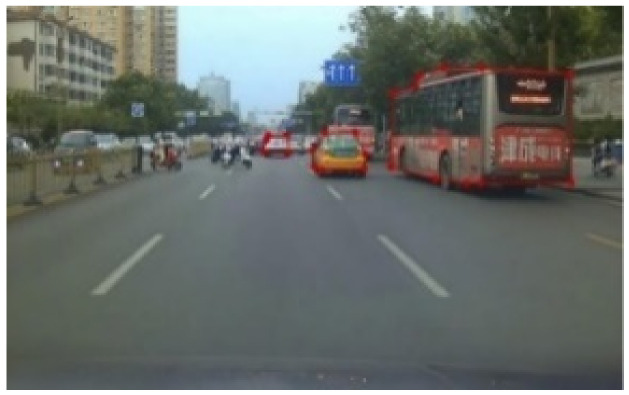
Polygon vehicle labeling.

**Figure 8:**
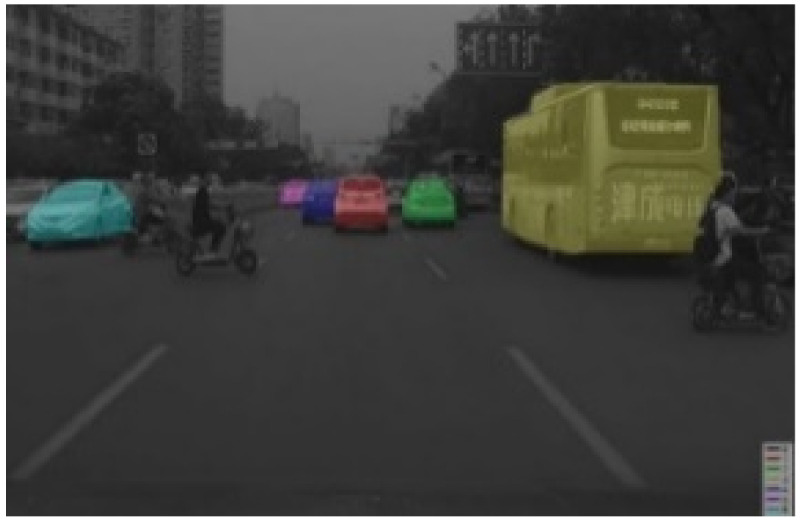
Instance segmentation annotation.

**Figure 9:**
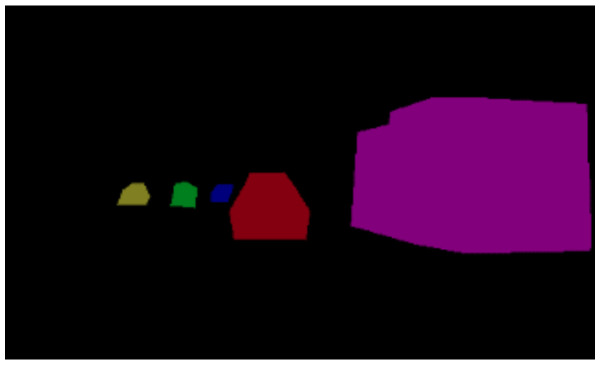
Mask labeling of instance segmentation.

Labelme software (https://github.com/wkentaro/labelme, accessed on 1 March 2020) uses polygon boxes to label target objects. We used Labelme software to label the entire shape of the vehicles, as shown in Figure 7. The effect diagrams of instance segmentation of vehicles on the road are shown in Figure 8 and Figure 9.

The vehicle dataset contained two folders, which were image and groundtruth. The image folder was a set of pictures with 1280 × 720 pixels, and the groundtruth folder was a set of segmentation mask pictures for vehicle instances.

### 2.3. Vehicle Lane-Changing Detecting Algorithm

**Figure 10:**
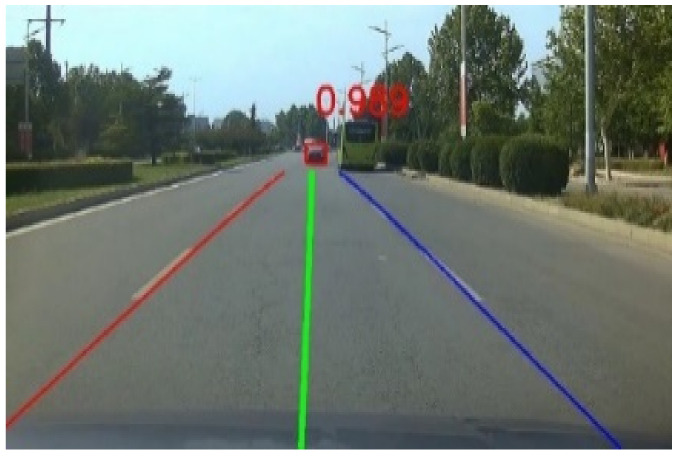
Vehicle and lane detection results of a frame.

Combining the improved vehicle detection network and the end-to-end lane detection network in series [23], the integrated outputs were obtained, as shown in Figure 10. We proposed a lane-changing detection method based on the first-person perspective, which can discriminate the target vehicle’s lane-changing behavior and obtain a higher detection accuracy.

The proportion of straight driving sections in the highway driving conditions is very large, and driving under good road conditions for a long time will make the driver tired and easily cause accidents. Therefore, it is necessary to inform the driver of the lane-changing behavior of the preceding vehicles under highway conditions to reduce the accident rate. The lane-changing detection algorithm made the following assumptions: (1) the driver’s ego-vehicle was stable without obvious lateral displacement; (2) the driving section was a straight section.

**Figure 11:**
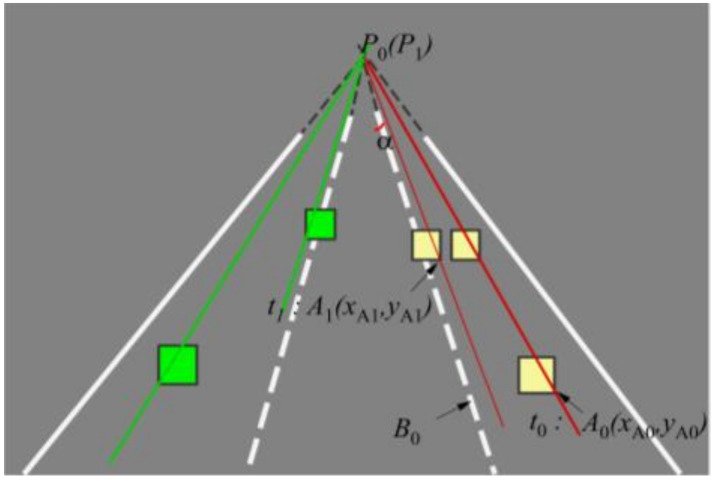
Schematic diagram of the lane change-detecting algorithm for the preceding vehicles.

According to the perspective principle, two parallel lane lines will intersect at a point in the distance. If two other parallel lines are added between the two parallel lines, because the fan-shaped area in the two lane lines eventually converge to a point, the other two parallel lines eventually converge to the same intersection. Thus, the four parallel lanes will eventually converge to a point *P*_0_ (*P*_1_) in the distance. As shown in Figure 11.

**Figure 12:**
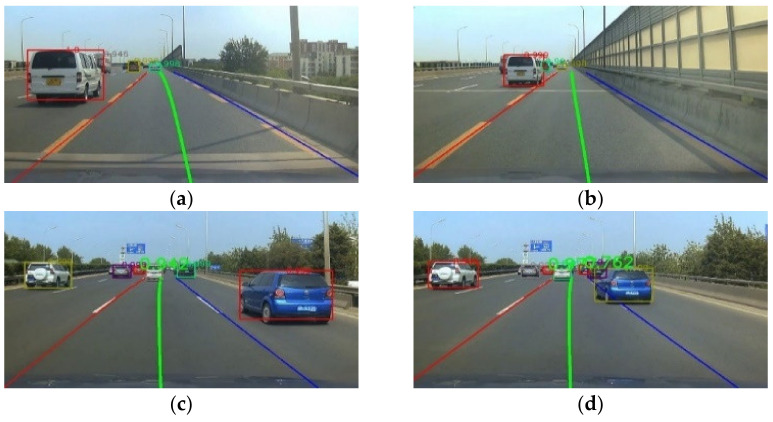
Selection of vehicle observation points.(**a**) detection result of the white van. (**b**) detection result of the white van. (**c**) detection result of the blue vehicle. (**d**) detection result of the blue vehicle.

The middle lane in Figure 12 was the driving lane of the ego-vehicle, and the other two lanes were adjacent lanes on the left and right. All lane lines intersected at a point *P*_0_. The lower corner points on the outside of the target vehicle detection frames were selected as the mark points connected with the point *P*_0_. The reason for choosing the lower corner points on the outside of the detection frames as the marking points was as follows.

Figure 12a,b show the detection characteristics of the white van on the adjacent left lane. It could be seen that the lower left corner of the white van detection frame was close to the body, and there was basically no offset. As the distance between the target vehicle and the ego-vehicle increased, the information on the right side of the target vehicle was gradually reduced and compressed, resulting in a shift in the lower right corner point to the left. Figure 12c,d show the detection results of the blue target vehicle on the adjacent right lane line. Therefore, the lower right corner of the detection frame should be selected as the target vehicle marking point to avoid the relative deviation in the marking point caused by the change in relative displacement of the target vehicle and ego-vehicle.

The lower right corner of the target vehicle in the right adjacent lane at time t_0_ was marked as *A*_0_, and the pixel coordinates of *A*_0_ in Figure 11 were (*x*_A0_, *y*_A0_). The target vehicle changed lane to the left, the lower right corner of the target vehicle at time t_1_ was marked as *A*_1_ (*x*_A1_, *y*_A1_), and the yellow box on the right side indicated the position where the target vehicle did not change lane. In Figure 11, a point on the right lane line of the ego-vehicle was marked as *B*_0_, and ∠*A*_0_*P*_0_*B*_0_ was set to the angle *α*. The angle *α* between frames would change, and the rate of change of the angle *α* between frames was defined as *β*_t_. For time t (frame):

$$\beta_{t}=\frac{\alpha_{t}-\;\alpha_{t-1}}{\alpha_{t}}$$

where *α*_t_ is the angle *α* at time t, *α*_−1_ is the angle *α* of the previous frame at time t, and *β*_t_ is the inter-frame change rate.

As the two premise assumptions of the lane-changing detection algorithm were met, the road intersection *P*_0_ of adjacent frames and *P*_1_ (the next frame intersection) approximately coincided. If the target vehicle with the yellow detection frame on the right was driving along the parallel direction of the lane, at time *t*_1_ (the next frame), the lower right corner of the target vehicle detection frame should be near the straight line *P*_1_*A*_0_.

If the target vehicle on the right made a lane-changing to the ego-vehicle lane, the lower right corner point at this moment was *A*_1_ (*x*_A1_, *y*_A1_), which should be on the left side of the line *P*_1_*A*_0_, and the corresponding angle *α* also reduced.

When the target vehicle changed lanes, the inter-frame angle α would decrease. As the target vehicle lane-changing was a continuous behavior, the inter-frame change rate should remain at a certain negative value. According to this, the lane-changing behavior could be determined according to the change law of the inter-frame change rate. The actual measurement found that the rate of change between frames was negative in most cases, but positive in a few cases. In order to suppress the influence of the detection fluctuation of some frames, a measure was taken to accumulate the inter-frame change rate. If the detection misalignment of the previous frame caused the inter-frame change rate to fluctuate greatly, the accurate detection of the next frame of the picture would cause the inter-frame change rate to fluctuate in the opposite direction, which would largely suppress the detection fluctuation. We designed a data pool (array) with 5 elements. The detection result of each frame of the picture was the inter-frame change rate, which is rolled and stored in sequence, summing the elements of the array and taking the opposite number, and comparing it with the adjustable parameter threshold *ρ*. If it was greater than *ρ*, it was regarded as a lane-changing; otherwise, no lane change had occurred. For a specific calculation example, see the introduction in Section 4.

## 3. Results

### 3.1. The Improved Effects of Vehicle Detection Network Training

Based on the improved Mask-RCNN, the vehicle targets were detected and the bounding boxes of the vehicle targets were drawn. As the vehicle dataset we have established was relatively small, over-fitting problems might easily occur if we directly performed long-term training based on this sample. Therefore, 20 epochs of pre-training were performed on the COCO train2017 dataset using Resnet50 architecture. When training vehicles, a data augmentation method was used to suppress the possible overfitting of the network.

**Figure 13:**
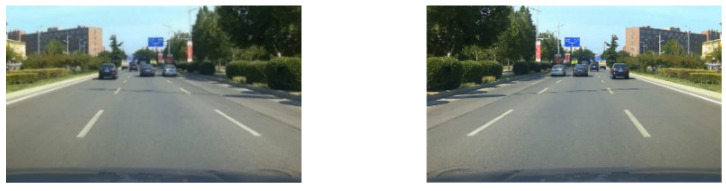
The random horizontal flipping.

We made a random horizontal flip on the training data, and set the application probability to 0.5, as shown in Figure 13.

In order to suppress over-fitting and speed up training, we constantly adjusted the reasonable parameters during the training period. The final specific parameter settings are shown in Table 1, which refer to this link (https://haochen23.github.io/2020/06/fine-tune-mask-rcnn-pytorch.html, accessed on 1 June 2020).

The experimental environment was ubuntu 18.04, a 64-bit system, the deep learning framework was Pytorch, the hardware was an Nvidia RTX 2070s, and the Python version was 3.6. We adopted the mean Average Precision (mAP) and Inference time (It) as the evaluation indexes.

**Figure 14:**
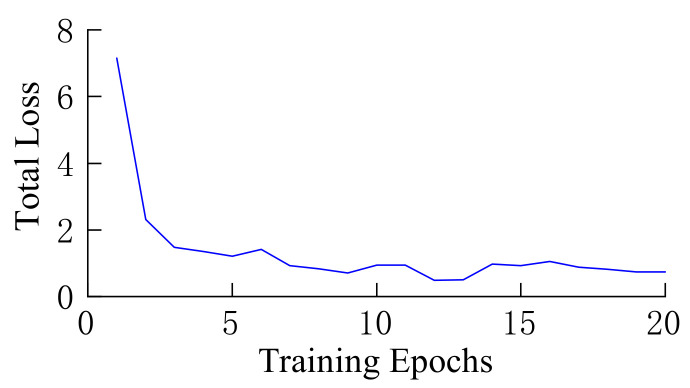
Loss function decline chart.

Figure 14 shows a graph of loss function decline after pre-training based on our self-built dataset retraining. As the training period increased, the loss function decreased significantly, indicating the effectiveness of model training.

**Figure 15:**
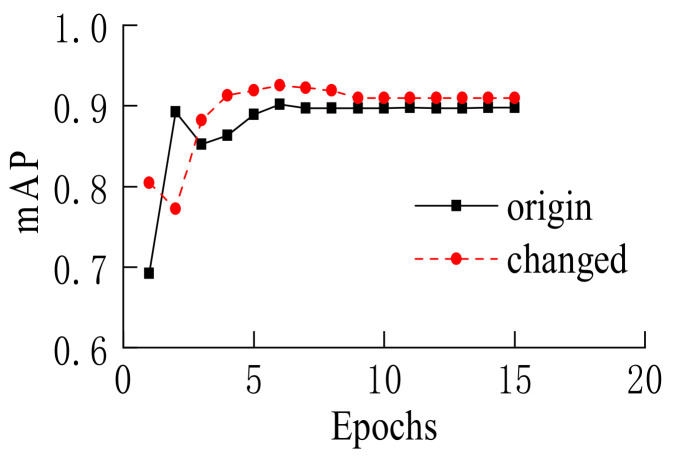
mAP of 0.5IoU.

Figure 15 shows a comparison of two anchor frame settings for the mAP indicator of the test set. The solid black line shows the results of the original Mask-RCNN network setting. The dashed red line shows the results of adding a new set of height-width ratios of anchor frame. It could be seen that after adding a new set of anchor frames that were more suitable for the proportion of vehicle detection frames, the mAP of 0.5IoU was significantly improved compared to the original setting, with an improvement rate of about 2.6%. Because the model was first trained on the CoCo2017 dataset for a long time, the value of mAP was relatively high even if it was not trained on the self-built dataset. After adding a set of anchor frames that were more suitable for the proportion of the vehicle target detection frame, although the convergence speed of the model had not been significantly improved, the mAP index had been significantly increased, which improved the accuracy of vehicle target detection.

**Figure 16:**
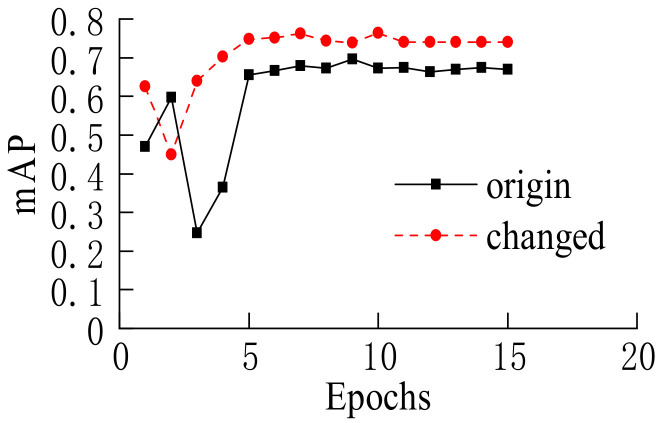
mAP of 0.75IoU.

It could be seen from Figure 16 that after a new set of anchor frames that were more suitable for the proportion of vehicle detection frame, the mAP of 0.75IoU was significantly improved compared to the original setting, with an increase of about 10.5%, which greatly improved the accuracy of vehicle target detection.

**Figure 17:**
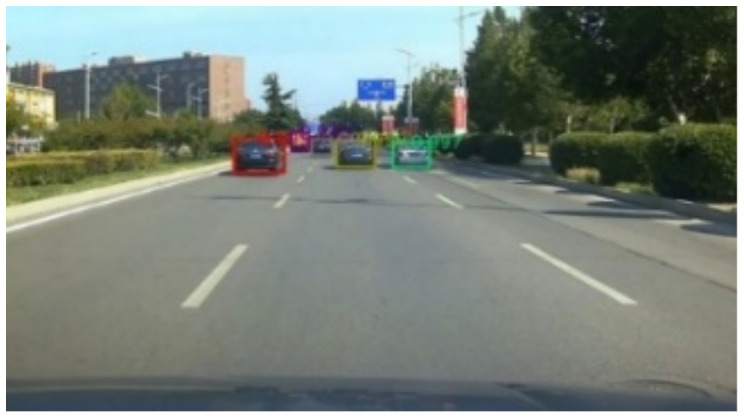
Detection results.

**Figure 18:**
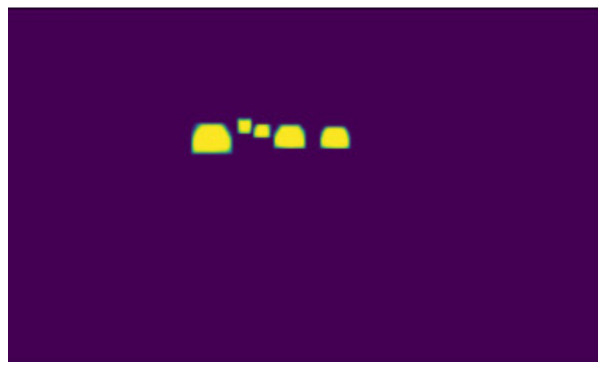
Output masks of detection network.

Figure 17 shows the output results of the bounding box of network vehicles. The numbers on the upper side of the vehicle box indicate the accuracy of the target being detected as a vehicle. The accuracy rates from left to right were 0.999, 0.943, 0.993, 0.999, and 0.997, respectively. Figure 18 shows the output results of the network vehicle mask. It could be seen that the network had a good detection effect on vehicle targets, and the frames were closely enveloped with the vehicles.

As shown in Table 2, we added a set of anchor frames and performed inference tests on the dataset. The average inference time increased from 0.109 s in the original network to 0.117 s, and the time consumption increased by about 7%. The time-consumption increase was not very obvious, and it could still meet the timeliness requirement of the lane-changing behavior detection.

### 3.2. The Angle α between Frames during Vehicle Lane-Changing

**Figure 19:**
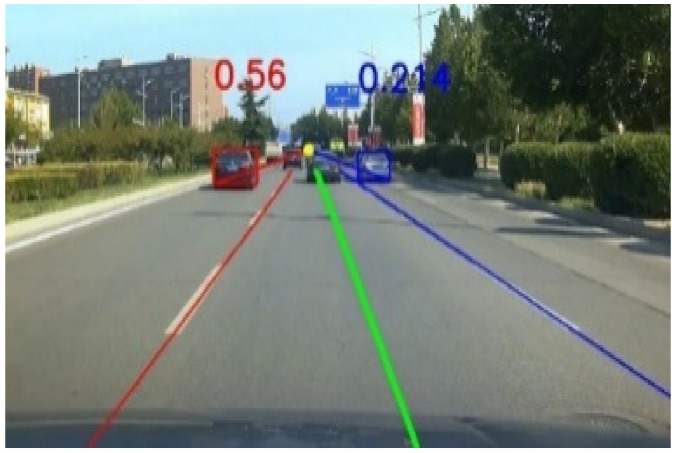
Diagram of lane corner and angle *a*.

As shown in Figure 19, the road information of the target vehicles in the left and right lanes on both sides of the ego-vehicle lane is displayed. The yellow dot showed the distant intersection point *P*_0_ of the two lane lines of the ego-vehicle, and the red detection box surrounded the vehicle closest to the ego-vehicle on the left lane line. The angle formed by the lower left corner of the red detection frame, the far corner point *P*_0_ of the two lane lines of the ego-vehicle, and the left lane line was 0.560 rad. The corresponding angle formed by the lower right corner of the blue detection frame on the right was 0.214 rad.

**Figure 20:**
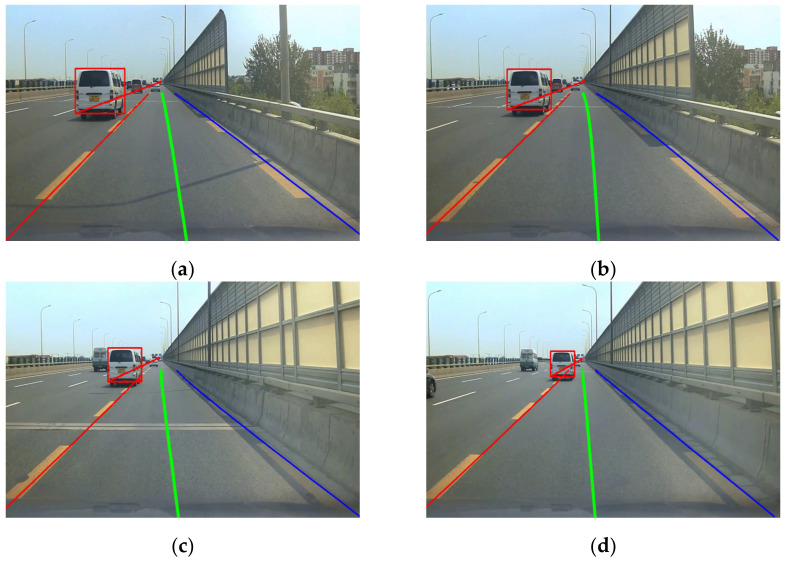
Target vehicle angle α changing with the target vehicle lane-changing. (**a**) First frame. (**b**) Third frame. (**c**) Fifth frame. (**d**) Seventh frame.

Figure 20 shows the change in angle α when the target vehicle in the left lane changed lane. The angles formed by the lower left corner of the red detection frame, the far corner point *P*_0_ of the two lane lines of the ego-vehicle, and the left lane line were 0.407, 0.380, 0.318, and 0.197 rad, respectively, from Figure 20a–d. It could be seen that as the left target vehicle changed lane to the ego-vehicle lane, the angle α decreased gradually.

## 4. Discussion

Before designing the lane-changing warning system, it was necessary to produce statistics on the change in *α* in each frame of the picture and the value of the inter-frame change rate *β*_t_ during the course of the lane-changing behavior to find the certain rule, and then set the corresponding judgment index through the rule.

**Figure 21:**
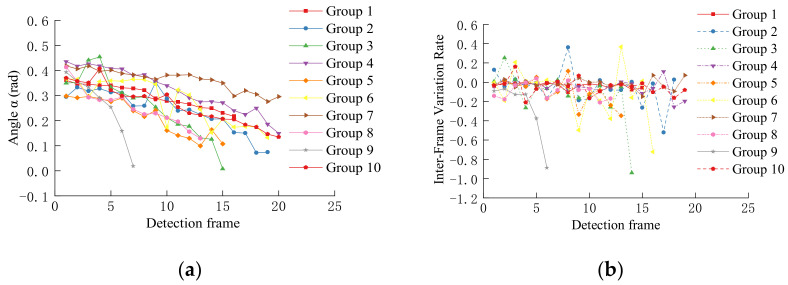
(**a**) *α* in target vehicle lane-changing behavior. (**b**) *β*_t_ caused by target vehicle lane-changing.

Figure 21a shows a graph of changes in angle α of the target vehicle lane-changing behavior at the left and right adjacent lanes sampled in ten groups (data source: China Automotive Engineering Research Institute), and the abscissa is the number of frames. The frame rate of the data source video was 25 frames per second. The total time of the vehicle detection network and the lane detection network was about 200 ms. The 25 frames-per-second video was taken every 6 frames for analysis. It could be seen that as the target vehicle gradually changed lane to the ego-vehicle lane, the angle α gradually decreased and approached zero. Because the intensity of the lane-changing was different, the length of time from the beginning to the end of a lane-changing was also different. It could be seen that some of the curves fell faster (such as the ninth group) and took up less time frame. This was because the target vehicle had performed a fast lane-changing behavior.

Figure 21b shows the variation statistics of inter-frame variation rate *β*_t_ with the lane-changing of the target vehicle. During the continuous lane-changing of the target vehicle, *β*_t_ remained negative in most cases. The positive value appeared because there was an error in the lane line detection of a certain frame due to interference, which resulted in a large deviation in the point *P*_0_, or a misalignment in the vehicle detection frame.

According to the method in Section 2.3, a method of accumulating the inter-frame variation rate *β*_t_ was adopted to suppress the influence of the detection fluctuation of some frames. Through the above ten groups of lane-changing video data analysis, the lane-changing threshold *ρ* = 0.18 was set, and the value greater than the threshold was regarded as the target vehicle lane-changing. Based on this setting, the accuracy rate of the lane-changing behavior detection for 55 groups of target vehicles was 94.5%. We found that the cause of the loss was the missing slow lane-changing of some target vehicles. If the value of *ρ* was increased, the false alarms that the target vehicle does not change lane could be further suppressed. If the value of *ρ* was lowered, the lane-changing behavior of the target vehicle could be detected more sensitively.

The link to the program codes and dataset for target vehicle detection and lane-changing recognition used in this paper is https://www.aliyundrive.com/s/ydaqAXs52zh, accessed on 15 April 2022. Please download the PC desktop app from the website www.aliyundrive.com, and install Aliyun disk on your PC. Click the link, save the files to your own cloud disk, and then download the cloud disk to your PC.

## 5. Conclusions

In order to improve the driving safety of vehicles that are not equipped with a lane-changing warning driving assistance system at a lower cost, we used deep learning image processing technology and realized the research and development of the function of the lane-changing warning system based on a monocular camera. We made full use of the accuracy and real-time characteristics of the deep neural network to detect vehicle targets, and improved the Mask R-CNN network for vehicle target detection, the accuracy of vehicle target detection was improved, and the system could meet the real-time requirement. A vehicle lane-changing detection algorithm based on the first-person perspective was proposed, a high accuracy rate had been achieved, and a highly time-efficient, low-cost, and high-accuracy lane-changing warning function had been realized. In the future, we will consider further optimizing the target detection network structure to reduce the time-consuming detection; we will conduct warning research on vehicle lane-changing behavior in curve situations.

## Figures and Tables

**Table 1:** Setting parameters of vehicle detection network.

Parameters	Descriptions	Values
*L*_r_	Learning rate	0.005
*I*_c_	Impulse constant	0.9
*W*_d_	Weight decay	0.0005
*N*_t_	Data Number of threads	4
*I*_p_	Iteration periods	20
*R*	Ratio of training set to validation set	4:1

**Table 2:** Comparisons of time consumption before and after improvement.

Configuration	Average Inference Time/s	Average Number of Inference Frames
Original Mask R-CNN	0.109	9.174
Improved Mask R-CNN	0.117	8.547

## Data Availability

https://www.aliyundrive.com/s/ydaqAXs52zh, accessed on 15 April 2022.
